# Age-dependent difference in impact of fertility preserving surgery on disease-specific survival in women with stage I borderline ovarian tumors

**DOI:** 10.1186/s13048-018-0423-y

**Published:** 2018-06-29

**Authors:** Haiyan Sun, Xi Chen, Tao Zhu, Nanfang Liu, Aijun Yu, Shihua Wang

**Affiliations:** 1 Department of Gynecologic Oncology, Zhejiang Cancer Hospital, 1 Banshan East Road, Zhejiang, 310022 Hangzhou China; 2 Department of Gynecology, The First People’s Hospital of Aksu, Aksu, China; 3 Department of Cancer Biology, Wake Forest School of Medicine, Winston Salem, NC 27157 USA

**Keywords:** Ovarian cancer, Borderline ovarian tumor, Fertility preserving surgery, Survival, Age, Histology, Stage

## Abstract

**Background:**

This study was to determine age-specific impact of fertility preserving surgery on disease-specific survival in women with stage I borderline ovarian tumors (BOTs). Patients diagnosed during 1988–2000 were selected from The Surveillance, Epidemiology, and End Results (SEER) database. The age-specific impact of fertility preserving surgery and other risk factors were analyzed in patients with stage I BOTs using Cox proportion hazard regression models. Data from our hospital were collected during 1996–2017 to determine the prevalence of patients who had undergone fertility preserving surgery.

**Results:**

Of a total 6295 patients in the SEER database, this study selected 2946 patients with stage T1 BOTs who underwent fertility preserving or radical surgery. Their median age at diagnosis was 45.0 years and the median follow-up time was 200 months. Fertility preserving surgery was performed in 1000/1751 (57.1%) patients < 50 years and in 1,81/1195 (15.1%) patients ≥50 years. Fertility preserving surgery was significantly associated with worse disease-specific survival only in patients ≥50 years. Increased age, stage T1c and mucinous histology were risk factors for overall patients or patients ≥50 years, but not for < 50 years. Data from our hospital showed that fertility preserving surgery was performed in 53.9 and 12.3%patients < 50 and ≥ 50 years with stage I disease, respectively.

**Conclusion:**

Fertility preserving surgery is safe for women < 50 years with early staged BOTs, but it may decrease disease-specific survival in patients ≥50 years. Conservative surgery is performed at a relatively high rate in patients ≥50 years.

**Electronic supplementary material:**

The online version of this article (10.1186/s13048-018-0423-y) contains supplementary material, which is available to authorized users.

## Background

Borderline ovarian tumors (BOTs) are histologically characterized as atypical epithelial proliferation without the presence of stromal invasion [1]. Serous and mucinous BOTs are the two major histological types [2]. These tumors have a low malignant potential to spread beyond the ovary with peritoneal involvement [1] and have an excellent prognosis [3–5]. This disease accounts for 10–15% of all epithelial ovarian cancers [6]. Compared to invasive epithelial ovarian cancers, BOTs occur more commonly, at an early stage, in women of childbearing ages [7].

The majority of BOTs are managed with surgery alone. Fertility preserving surgery is widely adopted for patients who have early-stage tumor development and a desire for fertility. Current consensus states that fertility preserving surgery is associated with an increased risk of recurrence [8–12]. Data from ours and other groups showed that certain styles of fertility preserving surgery may have a higher risk of recurrence than the others [13–15]. However, fertility preserving surgery was not shown to compromise overall survival in these patients [16–19].

Due to excellent prognosis, many patients with BOTs die due to other diseases. Overall survival is the end-point commonly used in previous studies to determine the impact of fertility preserving surgery; however, this may not accurately reflect the outcome of the surgery. Very few studies have investigated the impact of fertility preserving surgery on disease-specific survival [9]. Fertility preserving surgery has been shown age-dependent differences in its impact on recurrence free survival and other clinical outcomes in patients with BOTs [20]. Using a large population from a publicly available database, the objective of this study was to examine the age-specific impact of fertility preserving surgery on disease-specific survival in women with stage I BOTs.

## Methods

The data for this study was obtained from the Surveillance, Epidemiology, and End Results (SEER) database maintained by the National Cancer Institute. This database collects information of cancer patients, which covers approximately 28% of the total US population. The SEER program statistical analysis software package (SEER*Stat version 8.3.4) was used to extract data from SEER18 Regs Research Data + Hurricane Katrina Impacted Louisiana Cases, Nov 2016 Sub (1973–2014 varying) [21]. BOTs in the SEER database between 1988 and 2000 were identified based upon the following histopathology codes: serous 8442–1, 8451–1 and 8462–1; and mucinous 8472–1, 8473–1 [22, 23].

Only patients with stage I BOTs with a record of survival times were included in this study. The status of oophorectomies and hysterectomies were quarried from codes in the site-specific surgery (1983–1997) and RX Summ–Surg Prim Site (1998+) (Additional file 1: Table S1). Fertility preserving surgery refers to preservation of the uterus and at least one side of a functional ovarium. This study thus defined the surgery as removal of the tumor or a unilateral oophorectomy without a hysterectomy. Radical surgery was defined as bilateral salpingo-oophorectomy with or without hysterectomy. Women were excluded if they did not receive surgery, their surgical status or survival time was unknown, or other surgical approaches were performed (Additional file 1: Table S1). The flow chart shows the detailed procedure for selecting patients (Additional file 1: Figure S1).

Variables extracted from the database were patients’ demographics (age at diagnosis, ethnicities, marital status), surgery information (oophorectomy, hysterectomy, lymphadenectomy), tumor information (size, histology, stage), follow-up time and disease-specific death. Tumor stages were evaluated based on the American Joint Committee on Cancer (AJCC) 3rd staging classification [24].

To understand age-specific prevalence of fertility preserving surgery, women diagnosed with BOTs in Zhejiang Cancer Hospital during the year 1996–2017 were also included in this study. Tumor stages were evaluated based upon of the International Federation of Gynecology and Obstetrics (FIGO) 2014 classification system [25]. Stage T1 defined in AJCC 3rd is the same as stage I in FIGO 2014, except that stage Ic in FIGO 2014 is further divided into Ic1, Ic2 and Ic3 stages. The inclusion and exclusion criteria for these patients has been described previously [15].

Data were analyzed using SAS software V9.3 (SAS Institute, Inc., Cary, NC.). The ordinal/categorical data were examined using the χ^2^ test. Univariate or multivariate Cox proportional hazards models were used to determine the impacts of fertility preserving surgery and other risk factors on disease-specific survival. The Kaplan-Meier survival curves were generated and their significant differences were analyzed by log-rank tests. Two-sided *P* values less than 0.05 were considered statistically significant.

## Results

A total of 6295 women with BOTs were initially identified from the SEER database. Based on our inclusion and exclusion criteria, a total of 2946 cases with stage I BOTs were included in this study. The detailed demographic information and pathoclinical features are listed in Table 1. The mean age of these patients was 47.1 ± 17.0 years with a median age of 45.0 years (range 10–96 years). The median follow-up time was 200 months (range 1–323 months). Within this population, 59.4% (*n* = 1751) were <  50 years old and 40.6% (*n* = 1195) were ≥ 50 years. Most patients (85.0%) studied were Caucasian. The majority of BOTs were diagnosed at stage T1a (79.3%). Fertility preserving surgery was performed in 1181 (40.1%) patients. Hysterectomy and recorded lymphadenectomy were performed in 1374 (47.6%) and 341 (11.4%) patients, respectively. At the end of the follow-up year, 70 (2.4%) patients died from this disease.

**Table 1:** Demographic and pathoclinical features of BOT patients

Variables		Overall (*n* = 2946)	< 50 (n = 1751)	≥ 50 (n = 1195)	*P* value
Age (years)					
Median (range)		45.0 (10–96)	36.0 (10–49)	64.0 (50–96)	
Mean ± SD		47.1 ± 17.0	35.3 ± 8.6	64.3 ± 10.2	
Race					
	White	2505 (85.0)	1463 (83.6)	1042 (87.2)	0.0049
	Black	170 (5.8)	102 (5.8)	68 (5.7)	
	Others	271 (9.2)	186 (10.6)	85 (7.1)	
Histology					
	Serous	1646 (55.9)	961 (54.9)	685 (57.3)	0.1905
	Mucinous	1300 (44.1)	790 (45.1)	510 (42.7)	
Marital status					
Single*		1268 (43.0)	736 (42.0)	532 (44.5)	0.3188
	Married	1560 (53.0)	940 (53.7)	620 (51.9)	
	Unknown	118 (4.0)	75 (4.3)	43 (3.6)	
Lymphadenectomy					
	No	2602 (88.3)	1575 (90.0)	1027 (85.9)	0.0004
	Yes	336 (11.4)	169 (9.6)	167 (14.0)	
	Unknown	8 (0.3)	7 (0.4)	1 (0.1)	
AJCC stage					
	T1a	2337 (79.3)	1407 (80.4)	930 (77.8)	0.0582
	T1b	177 (6.0)	90 (5.1)	87 (7.3)	
	T1c	281 (9.6)	171 (9.8)	110 (9.2)	
	T1x	151 (5.1)	83 (4.7)	68 (5.7)	
Tumor size					
	≤ 5 cm	425 (40.6)	244 (40.3)	181 (40.9)	0.8402
	> 5 cm	622 (59.4)	361 (59.7)	261 (59.1)	
Hysterectomy	No	1572 (53.4)	1138 (65.0)	434 (36.3)	< 0.0001
	Yes	1374 (47.6)	613 (35.0)	761 (63.7)	
Fertility preserving surgery					
	No	1765 (59.9)	751 (42.9)	1014 (84.9)	< 0.0001
	Yes	1181 (40.1)	1000 (57.1)	181 (15.1)	
Laterality					
	Unilateral	1092 (37.1)	646 (36.9)	446 (37.3)	0.9652
	Bilateral	1253 (42.5)	748 (42.7)	505 (42.3)	
	Unknown	601 (20.4)	357 (20.4)	244 (20.4)	
Death	No	2876 (97.6)	1735 (99.1)	1141 (95.5)	< 0.0001
	Yes	70 (2.4)	16 (0.9)	54 (4.5)	
Follow-up time (months)					
Median (range)		200 (1–323)	217 (1–323)	176 (1–323)	
Mean ± SD		194.0 ± 72.0	215.0 ± 59.2	163.4 ± 77.8	< 0.0001

*including never married, divorced, widowed. Abbreviations: AJCC, American Joint Commission on Cancer; T1x, T1 undefined

The characteristics of patients in two age groups (< 50 and ≥ 50 years) are presented in Table 1. Compared to patients < 50 years, patients ≥50 years underwent fertility preserving surgery less frequently (15.1% vs 57.1%, *P* < 0.0001). A higher proportion of them were Caucasian (87.2% vs 83.6*%, P* = 0.0049), underwent hysterectomy (63.7% vs 35.0%, *P* < 0.0001) and lymphadenectomy (14.0% vs 9.6%, *P* = 0.0050). They had a higher rate of disease-specific death (4.5% vs 0.9%, *P* < 0.0001), but a shorter mean follow-up time (163.4 ± 77.8 vs 215.0 ± 59.2 months, *P* < 0.0001).

The features of patients were compared between those who underwent fertility preserving surgery vs. radical surgery. Of the entire population studied, including both age groups, married patients and patients with serous tumors at stage T1b or T1c were less likely to undergo fertility preserving surgery. Patients receiving fertility preserving surgery were less likely to undergo lymphadenectomy. Caucasian patients, both in the entire population, as well as in the < 50 age group were less likely to undergo fertility preserving surgery (Table 2).

**Table 2:** Features of patients who underwent fertility preserving surgery (Yes) or radical surgery (No)

Variables		Total (n = 2946)			< 50 (n = 1751)			≥ 50 (n = 1195)		
Fertility preserving surgery		Yes	No	*P* values	Yes	No	*P* values	Yes	No	*P* values
Race										
	White	964 (81.6)	1541 (87.3)	< 0.0001	806 (80.6)	657 (87.5)	< 0.0001	158 (87.3)	884 (87.2)	0.0952
	Black	75 (6.4)	95 (5.4)		60 (6.0)	42 (5.6)		15 (8.3)	53 (5.2)	
	Other	142 (12.0)	129 (7.3)		134 (72.0)	52 (6.9)		8 (4.4)	77 (7.6)	
Marital status										
	Single*	580 (49.1)	688 (39.0)	< 0.0001	487 (48.7)	249 (33.2)	< 0.0001	93 (51.4)	439 (43.3)	0.0220
	Married	555 (35.6)	1005 (56.9)		477 (47.7)	463 (61.6)		78 (43.1)	542 (53.4)	
	Unknown	46 (3.9)	72 (4.1)		36 (3.6)	39 (5.2)		10 (5.5)	33 (3.3)	
Histology										
	Serous	615 (52.1)	1031 (58.4)	0.0007	514 (51.4)	447 (59.5)	0.0007	101 (55.8)	584 (57.6)	0.6533
	Mucinous	566 (47.9)	734 (41.6)		486 (48.6)	304 (40.5)		80 (44.2)	430 (42.4)	
AJCC stage										
	T1a	1000 (84.7)	1337 (75.6)	< 0.0001	850 (85.0)	557 (74.2)	< 0.0001	150 (82.9)	780 (76.9)	0.0008
	T1b	22 (1.9)	155 (8.8)		21 (2.1)	69 (9.2)		1 (0.6)	86 (8.5)	
	T1c	101 (8.6)	180 (10.2)		86 (8.6)	85 (11.3)		15 (8.3)	95 (9.4)	
	T1x	58 (4.9)	93 (5.3)		43 (4.3)	40 (5.3)		15 (8.3)	53 (5.2)	
Hysterectomy										
	No	1181 (100)	391 (22.2)	< 0.0001	100 (100)	138 (18.4)	< 0.0001	181 (100)	253 (25.0)	< 0.0001
	Yes	0 (0)	1374 (77.8)		0 (0)	631 (81.6)		0 (0)	761 (75.0)	
Tumor size										
	<=5	166 (40.3)	259 (40.8)	0.8731	139 (39.8)	105 (41.0)	0.7686	27 (42.9)	154 (40.6)	0.7396
	> 5	246 (59.7)	376 (59.2)		210 (60.2)	151 (59.0)		36 (57.1)	225 (59.4)	
Lymphadenectomy										
	No	1085 (91.9)	1517 (86.0)	< 0.0001	1032 (61.3)	652 (38.7)	0.0001	161 (89.0)	866 (85.4)	0.4254
	Yes	91 (7.8)	245 (13.9)		80 (44.7)	99 (55.3)		20 (11.0)	147 (14.5)	
	Unknown	5 (0.4)	3 (0.2)		5 (71.4)	2 (28.6)		0 (0)	1 (0.1)	
Laterality										
	Unilateral	426 (36.1)	666 (37.7)	0.6573	372 (37.2)	274 (36.5)	0.8792	54 (29.8)	392 (38.7)	0.0679
	Bilateral	510 (43.2)	743 (42.1)		422 (42.2)	326 (43.4)		88 (48.6)	417 (41.1)	
	Unknown	245 (20.7)	356 (20.2)		206 (20.6)	151 (20.1)		39 (21.6)	205 (20.2)	
Death	No	1162 (98.4)	1718 (97.1)	0.0253	994 (99.4)	741 (98.7)	0.1113	168 (92.8)	973 (96.0)	0.0611
	Yes	19 (1.6)	51 (2.9)		6 (0.6)	10 (1.3)		13 (7.2)	41 (4.0)	

*including never married, divorced, widowed. Abbreviations: AJCC, American Joint Commission on Cancer; T1x, T1 undefined

Results of univariate and multivariate analysis of disease-specific survival in the whole population are presented in Table 3. Increased age (hazard ratio (HR) = 1.06, 95% confidence interval (CI): 1.04–1.08, *P* < 0.0001), stage T1c (vs T1a, HR = 2.42, 95% CI: 1.30–4.48, *P* = 0.0051) were significantly associated with worse disease-specific survival. Without controlling of other confounding factors, fertility preserving surgery (vs radical surgery, HR = 0.52, 95% CI: 0.30–0.88, *P* = 0.0142) was associated with improved disease-specific survival. The survival curves are presented at Additional file 1: Figure S2A and S2B. Multivariate analysis showed that increased age (HR = 1.06, 95% CI: 1.05–1.08, *P* < 0.0001), stage T1b (vs T1a, HR = 2.38, 95% CI: 1.05–5.39, *P* = 0.0369), stage T1c (vs T1a, HR = 3.00, 95% CI: 1.60–5.65, *P* = 0.0006) and mucinous histology (HR = 1.73, 95% CI: 1.06–2.83, *P* = 0.0285) were significantly associated with worse disease-specific survival, whereas fertility preserving surgery is not a factor significantly related to disease-specific death.

**Table 3:** Survival analysis of cancer specific survival in the whole population

Variables		Univariate		Multivariate	
		HR (95%CI)	*P* values	HR (95%CI)	*P* values
Age		1.06 (1.04–1.08)	< 0.0001	1.06 (1.05–1.08)	< 0.0001
AJCC stage				1	
	T1a	1			
	T1b	2.22 (1.00–4.92)	0.0503	2.38 (1.05–5.39)	0.0369
	T1c	2.42 (1.30–4.48)	0.0051	3.00 (1.60–5.65)	0.0006
	T1x	1.58 (0.63–3.98)	0.3318	1.45 (0.57–3.67)	0.4349
Histology					
	Serous	1		1	
	Mucinous	1.40 (0.88–2.24)	0.1600	1.73 (1.06–2.83)	0.0285
Race					
	White	1			
	Black	0.75 (0.24–2.40)	0.6309		
	Other	0.57 (0.13–1.35)	0.1479		
Marital status					
	Single	1			
	Married	0.82 (0.52–1.32)	0.4191		
Unknown		0	0.9831		
Fertility preserving Surgery					
	No	1			
	Yes	0.52 (0.31–0.88)	0.0142		
Hysterectomy					
	No	1			
	Yes	1.04 (0.65–1.66)	0.8755		
Tumor size					
	<=5	1			
	> 5	1.47 (0.55–3.92)	0.4438		
Lymphadenectomy					
	No	1			
	Yes	0.77 (0.33–1.77)	0.5330		
	Unknown	0	0.9854		
Laterality					
	Unilateral	1			
	Bilateral	1.13 (0.767–1.93)	0.6432		
	Unknown	1.13 (0.59–2.14)	0.7208		

Abbreviations: AJCC, American joint commission on Cancer; T1x, T1 undefined

**Fig. 1:**
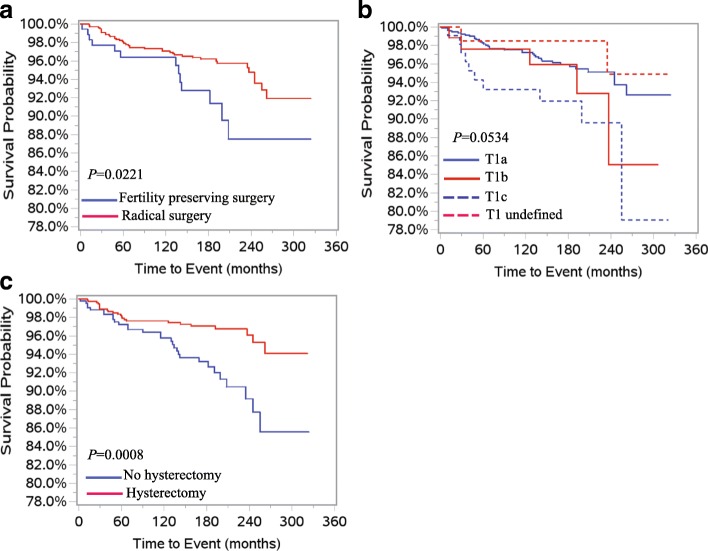
Kaplan-Meier survival curves for patients ≥50 with stage I borderline ovarian tumors. **a** Fertility preserving surgery vs radical surgery. **b** Sub-stages. **c** Hysterectomy status

We further preformed survival analysis for patients in < 50 and ≥ 50 age groups. In patients < 50 years old, only the undefined T1 stage (vs T1a, HR = 5.99, 95% CI: 1.59–22.60, *P* = 0.0082) was significantly associated with poorer disease-specific survival. No other significant risk factors were observed in these patients using univariate analysis. No risk factors were correlated with disease-specific survival using multivariate analysis (Table 4). In patients ≥50 years, univariate analysis showed that increased age (HR = 1.04, 95% CI: 1.01–1.07, *P* = 0.0063), fertility preserving surgery (HR = 2.04, 95% CI: 1.09–3.81, *P* = 0.0251), stage T1c (vs T1a, HR = 2.38, 95% CI: 1.18–4.78, *P* = 0.0151) and hysterectomy (HR = 0.41, 95% CI: 0.24–0.70, *P* = 0.0012) were risk factors significantly associated with disease-specific survival (Table 5). Disease-specific survival curves of the above risk factors are presented at Fig. 1a, b and c. Multivariate analysis showed that the increased age (HR = 1.04, 95% CI: 1.01–1.07, *P* = 0.0108), fertility preserving surgery (HR = 1.99, 95% CI: 1.059–3.77, *P* = 0.0253), stage T1c (HR = 2.87, 95% CI: 1.41–5.86, *P =* 0.0037) and mucinous histology (HR = 1.87, 95% CI: 1.07–3.27, *P =* 0.0278) were risk factors significantly associated with worse disease-specific survival (Table 5).

**Table 4:** Univariate survival analysis in patients of age < 50 years

Variables		HR (95%CI)	*P* values
Age		1.04 (0.98–1.11)	0.2070
Race			
	White	1	
	Black	0	0.9908
	Other	1.19 (0. 27–5.25)	0.8173
Marital status			
	Single*	1	
	Married	1.66 (0.58–4.79)	0.3467
	unknown	0	0.9920
Histology	Serous		
	Mucinous	1.20 (0.45–3.19)	0.7200
AJCC stage			
	T1a	1	
	T1b	4.12 (0.87–19.41)	0.0734
	T1c	3.07 (0.81–11.57)	0.0979
	T1x	5.99 (1.59–22.60)	0.0082
Fertility preserving surgery			
	No	1	
	Yes	0.46 (0.17–1.28)	0.1374
Hysterectomy			
	No	1	
	Yes	2.26 (0.84–6.07)	0.1061
Tumor size	≤ 5 cm	1	
	> 5 cm	0.75 (0.110–5.40)	0.7771
Lymphadenectomy	No	1	
	Yes	0	0.9922
	Unknown	0	0.9986
Lymph node number			
	1–10	1	
	> 10	0	0.9911
	Unknown	0	0.9906
Laterality	Unilateral	1	
	Bilateral	150 (0.44–5.12)	0.5183
	Unknown	2.23 (0.60–8.31)	0.2314

*including never married, divorced, widowed. Abbreviations: AJCC, American Joint Commission on Cancer; T1x, T1 undefined

**Table 5:** Survival analysis in patients ≥ 50 years

Variables		Univariate		Multivariate*	
		HR (95% CI)	*P* values	HR (95% CI)	*P* values
Age		1.04 (1.01–1.07)	0.0063	1.04 (1.01–1.07)	0.0108
Fertility preserving surgery					
	No	1		1	
	Yes	2.04(1.09–3.81)	0.0251	1.99 (1.05–3.77)	0.0347
AJCC stage					
	T1a	1		1	
	T1b	1.56 (0.61–3.96)	0.3531	2.30 (0.87–6.09)	0.0931
	T1c	2.38 (1.18–4.78)	0.0151	2.87 (1.41–5.86)	0.0037
	T1x	0.60 (0.14–2.49)	0.4793	0.58 (0.14–2.42)	0.4527
Histology					
	Serous	1		1	
	Mucinous	1.53 (0.90–2.62)	0.1175	1.87 (1.07–3.27)	0.0278
Race					
	White	1			
	Black	1.01 (0.32–3.24)	0.9879		
	Other	0.23 (0.03–1.68)	0.1476		
Marital status					
	Single*	1			
	Married	0.63 (0.37–1.08)	0.0915		
	unknown	N/A	0.9860		
Hysterectomy					
	No	1			
	Yes	0.41 (0.24–0.70)	0.0012		
Size (cm)					
	<=5	1			
	> 5	1.00 (1.00–1.01)	0.3384		
Lymphadenectomy					
	No	1			
	Yes	0.78 (0.33–1.82)	0.5639		
	Unknown	0	0.9888		
Laterality					
	Unilateral				
	Bilateral	1.10 (0.61–1.99)	0.7511		
	Unknown	0.91 (0.42–1.94)	0.7986		

*including never married, divorced, widowed. Abbreviations: AJCC, American Joint Commission on Cancer; T1x, T1 undefined

Data from our hospital showed that 255 women with BOTs underwent surgery from 1996 to 2017. The median age was 42 years (range 15–87). Among these patients, 108 (42.4%) had serous tumors and 118 (46.3%) had mucinous tumors. A total of 170 (66.7%) cases were stage I, with one case having an unknown age. Fertility preserving surgery was performed in 113 overall (44.3%) patients (Additional file 1: Table S2). The rate of fertility preserving surgery performed in these patients at stage I was further analyzed after dividing them into two age groups (< 50 and ≥ 50 years). Our result showed that 56/104 (53.9%) patients < 50 and 8/65 (12.3%) patients ≥50 underwent fertility preserving surgery. These two groups had other similar pathoclinical features (Table 6).

**Table 6:** Pathoclinical features of patients with stage I borderline ovarian tumor from Zhejiang Cancer Hospital

Variables		Age (yeas)		
		< 50	≥50	*P* values
Fertility preservation surgery				
	No	48 (46.1)	57 (87.7)	< 0.0001
	Yes	56 (53.9)	8 (12.3)	
Histology				
	Serous	43 (41.3)	27 (32.3)	0.9774
	Mucinous	52 (50.0)	39 (60.0)	
	Endometrioid	7 (7.7)	4 (6.1)	
	Clear cells	1 (1.0)	1 (1.5)	
FIGO stage				
	IA	70 (67.3)	42 (64.6)	0.9263
	IB	11 (10.6)	7 (10.8)	
	IC	23 (22.1)	16 (24.6)	
Tumor size (cm)				
	≤5	42 (40.4)	21 (32.3)	0.2908
	> 5	62 (59.6)	44 (67.6)	
Laterality				
	Unilateral	80 (82.7)	49 (76.9)	0.3574
	Bilateral	24 (17.3)	16 (23.1)	
Death				
	No	103 (99.0)	64 (98.5)	0.6938
	Yes	1 (1.0)	1 (1.5)	

## Discussion

With a sample size of 2946 patients and a median follow-up time of 200 months, this study examined age-specific impact of fertility preserving surgery on disease-specific survival in women with T1 BOTs. The main finding of this study was that fertility preserving surgery was significantly associated with worse disease-specific survival only in patients ≥50 years, but not in overall patients or patients < 50 years. Our results revealed an age-dependent difference in impact of fertility preserving surgery on disease-specific survival in these patients. This finding suggests that while conservative surgery may comprise survival in women ≥50 years, it is safe for patients < 50 years. Future studies with randomized clinical trials are warranted to verify this finding.

Previous studies have consistently shown that fertility preserving surgery may increase the risk of recurrence [11, 14, 15, 26]. Interestingly, the risk of recurrence was higher in younger patients with BOTs [9, 11, 18, 20]. Most of the recurrences showed no malignant transformation and were curable by a single surgery without compromising overall survival [9, 16–18]. Invasive carcinoma diagnosed in recurrences [9, 11, 18, 27–29] is the cause of cancer deaths [30]. A sub-analysis of the Arbeitsgemeinschaft Gynaekologische Onkologie (AGO) ROBOT study evaluated data from a total of 950 patients with BOTs. Their results showed that 66.7% of recurrent diseases were invasive carcinoma in patients ≥40 years, which dramatically contrasted with a recurrence of 12% of invasive carcinomas in patients < 40 years [20]. The increased incidence of invasive recurrent ovarian cancer in older patients may account for the reduced disease-specific survival after fertility preserving surgery.

This study is unable to address the molecular mechanism whereby fertility preserving surgery is associated with reduced disease-specific survival in patients ≥50 years. Akeson et al. [7] reported patients > 60 had significantly more aneuploid tumors. Aneuploidy was associated with an increased mortality of patients with BOTs [31]. Furthermore, BRAF, KRAS and other mutations, and ERBB2 overexpression/amplification were frequently observed BOTs [32–34]. It is unknown whether age-related changes in DNA ploidy and gene mutations play a role in increased invasive recurrence in older patients.

It is noted that as high as 15.1% patients ≥ 50 years with stage I BOTs underwent fertility preserving surgery in this selected population. Reports are still sparse regarding the prevalence of patients undergoing fertility preserving surgery within different age groups. Trillsch et al. reported that fertility preserving surgery was carried out in 53.2% (149/280) of patients < 40 years, 2.8% (19/670) of overall patients ≥40 years with BOTs [20]. It is speculated that a higher rate of conservative surgery was performed in their patients with stage I BOTs. Comparable to the result from the SEER database, data from our hospital showed 12.3% women ≥50 years with stage I disease underwent fertility preserving surgery. Women ≥50 years lose reproductive ability. Preservation of fertility is therefore not the primary objective when adopting conservative surgery in these patients. Conservative surgery brings less postoperative morbidities. Specific reasons older patients undergo conservative surgery remain unknown. Based upon the findings of this study, these patients may need extra attention after conservative surgery.

Our study also identified that increased age, a higher stage (T1c) and mucinous histology were significantly associated with decreased disease-specific survival in overall patients or patients ≥50. Using the same database, a previous study revealed that older age (≥ 50), higher stage and mucinous histology were associated with worse disease-specific survival in patients with stage I BOTs [23]. The tumor stage is a known prognostic factor for patients with BOTs [29]. Our results further revealed that higher stage (T1c) was significantly associated with poorer disease-specific survival in BOT patients at the early stage. Patients with mucinous BOTs were reported to have a worse prognosis compared with to patients with serous BOTs [31, 35]. The worse survival is partially explained by a higher incidence of invasive recurrent carcinoma in patients with mucinous BOTs. Karlsen et al. [9] found that 6 out of 7 invasive recurrences were patients with mucinous BOTs at FIGO stage I.

An earlier study identified 6017 cases of BOTs from the SEER database. Their results revealed that the lymph node involvement was not significantly associated with disease-specific survival after adjusting with FIGO stages [36]. No impact of lymph node involvement on overall survival in patients with BOT were also observed in other studies [37, 38]. Data from our work and the previous study [23] showed that lymphadenectomy were not a risk factor associated with disease-specific survival.

The use of this database has numerous limitations. Patients were included retrospectively and were not randomly assigned to a treatment. Detailed information of fertility preserving surgery is unavailable. Among patients with stage I disease, 41.6% (2118/5094) were excluded from the study due to unclear surgical information. Many important pathological features of the tumors, such as invasive implants, and micropapillary patterns, are unavailable in these patients. Ovarian cancer related blood biomarkers were not recorded in the SEER database. It is unknown whether there have been recurrences and the types of relapses may have occurred in these patients. The location of harvested lymph nodes are not defined and their numbers are missing in some patients. Many other limitations using the SEER database have been addressed in a previous study [23].

Use of the SEER database in this study had its strength in its relatively large sample size, long follow-up time, and particularly, relatively large number of disease-specific deaths. Using the same database, the previous study identified 4943 cases with stage T1 BOTs from the same database, and reported a total of 159 (3.2%) deaths in a median follow-up time of 187 months [23]. In contrast, the number of disease-specific deaths reported in previous studies was limited. A cohort included 1143 BOT patients with 1005 (87.9%) patients at FIGO stage I. During a median follow-up time of 49.9 months (range 3.5–99 months), only 7 (0.6%) patients I died of this disease [9]. In another study, a total of 151 patients were recruited. Among them, 87 (64.4%) patients were at FIGO stage I, and 113 patients (74.8%) had follow-up information. After a median follow-up time of 86 (range 0.1–432) months, 7 (6.2%) patients died of this disease [39]. A multi-center study included 457 patients with 390 (85.3%) at stage I. During a mean follow-up of 88.3 months, 9 (2%) patients died of this disease [40]. Leake et al. reported 13 (6.5%) disease-specific deaths in a cohort of 200 patients in a median follow-up time of 120 months [41].

## Conclusion

It is safe to perform fertility preserving surgery for women of child-bearing age with stage I BOTs. This surgery may increase the risk of disease-specific death for women of older ages (≥ 50 years). A relatively high proportion of patients (≥ 50 years) receive conservative surgery.

## Additional file

Additional file 1: **Table S1.** Codes used to define surgery styles. **Table S2.** Features of patients with borderline ovarian tumors from our hospital during 1996–2017. **Figure S1.** Flowchart of population selection. **Figure S2.** Kaplan-Meier survival curves for all patients with stage I borderline ovarian tumors. (DOCX 134 kb)

## Abbreviations

AJCC: American joint committee on cancer
BOTs: Borderline ovarian tumors
FIGO: International Federation of Gynecology and Obstetrics
SEER: The surveillance, epidemiology, and end results
