# Supplementary material for: Age-dependent difference in impact of fertility preserving surgery on disease-specific survival in women with stage I borderline ovarian tumors
Source: J Ovarian Res. 2018 Jun 29;11:54. doi: 10.1186/s13048-018-0423-y (PMC6025735; doi:10.1186/s13048-018-0423-y)
Supplement: Supplementary file 1 — Table S1. Codes used to define surgery styles. Table S2. Features of patients with borderline ovarian tumors from our hospital during 1996–2017. Figure S1. Flowchart of population selection. Figure S2. Kaplan-Meier survival curves for all patients with stage I borderline ovarian tumors. (DOCX 134 kb) [file 13048_2018_423_MOESM1_ESM.docx]

**Supplemental data**

Table S1. Codes used to define surgery styles

| Surgery (Oophorectomy) | Site specific surgery (1973-1997 varying detail by year and site) | RX Summ--Surg Prim Site (1998+) |
| --- | --- | --- |
| No surgery | 00 | 00 |
| **Fertility preserving surgery*** | 10 | 12, 13, 14, 15, 16 |
| **Radical surgery **** | 30, 40, 70 | 21, 22, 70, 71, 72, 73, 74 |
| Other surgical styles | 20, 50, 51, 52, 60, 80 | 10, 11,13, 14, 16, 20, 30, 31, 32, 61, 62, 63, 80, 90 |
| Unknown surgery or not | 90 | 99 |

* Removal of tumor or unilateral removal of ovary without hysterectomy.

** Bilateral salpingo-oophorectomy with or without hysterectomy.

Table S2. Features of patients with borderline ovarian tumors from our hospital during 1996-2017.

| Variables |  | n (%)* |
| --- | --- | --- |
| Age (years) |  |  |
|  | Mean ± SD | 44.2 ± 15.1 |
|  | Median | 42.0 (15-87） |
|  | < 50 | 164 (64.3) |
|  | ≥ 50 | 89 (34.9) |
| Histology |  |  |
|  | Serous | 108 (42.4) |
|  | Mucinous | 118 (46.3) |
|  | Other | 21 (8.2) |
| FIGO Stage |  |  |
|  | I | 170 (66.7) |
|  | II | 25 (9.8) |
|  | III & IV | 34 (13.3) |
| Fertility preserving surgery | |  |
|  | No | 141 (55.3) |
|  | Yes | 113 (44.3) |

* percentage may not 100% due to patients with unknown information were not presented.

Figure S1. Flowchart of population selection. Fertility preserving surgery: total removal of tumor or unilateral removal of ovary without hysterectomy; Radical surgery: bilateral salpingo-oophorectomy with or without hysterectomy.


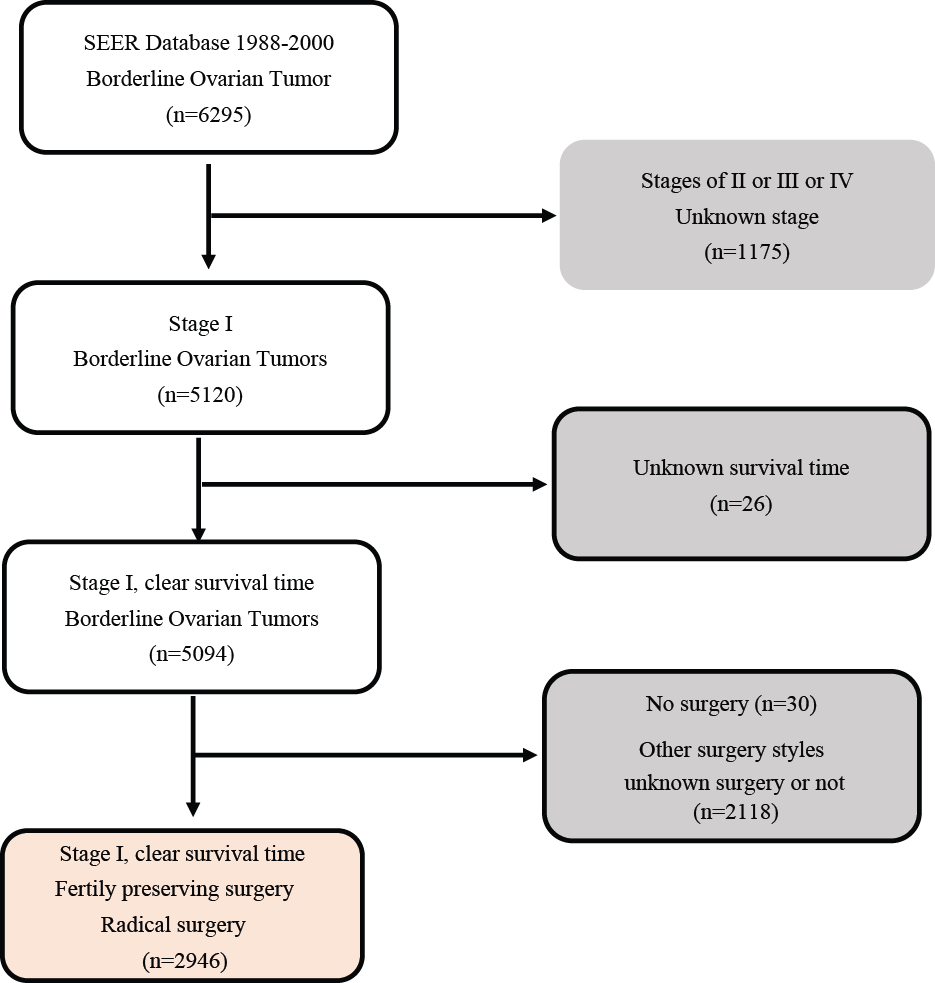


Figure S2.


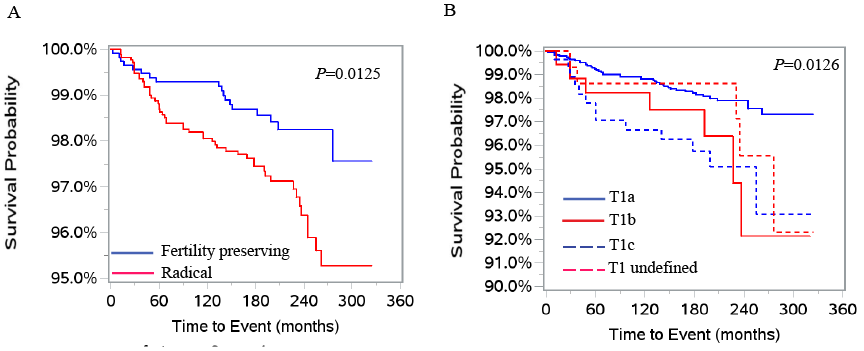


Figure S2. Kaplan-Meier survival curves for all patients with stage I borderline ovarian tumors.

(A) Surgical treatments (fertility preserving surgery vs radical surgery). (B) Sub-stages.
